# Response: “Nonspecific lower back pain in overhead athletes is multifactorial and is not solely determined by the type and intensity of the sport”

**DOI:** 10.17159/2078-516X/2026/v38i1a25502

**Published:** 2026-03-15

**Authors:** TP Moatshe

**Affiliations:** 1 Department of Physiotherapy, Sefako Makgatho Health Sciences University, Ga-Rankuwa, South Africa; 2 Physiotherapy Department, University of Pretoria, South Africa

I thank Dr Finsterer for his interest in our article, *“Prevalence and risk factors of non-specific low back pain among amateur overhead athletes in Gauteng Province,”* and for his comments highlighting the multifactorial nature of non-specific low back pain (NSLBP).

The primary aim of the study was to determine the prevalence of NSLBP and to identify selected sport-specific risk factors among amateur overhead athletes, rather than to provide an exhaustive assessment of all biological, psychological, and lifestyle contributors to NSLBP.^[1]^ This focused approach is consistent with existing sports medicine literature, which recognises NSLBP as multifactorial while supporting targeted investigation of sport-related contributors within defined populations.^[2,3]^

First, we agree that NSLBP may originate from multiple anatomical and systemic sources beyond the lumbar spine. However, the present study focused on NSLBP as it presents in physically active overhead athletes, where sport-related musculoskeletal loading and kinetic-chain function are clinically relevant. Participants with known neurological disease, systemic pathology, recent spinal or shoulder surgery, or other medical conditions likely to confound musculoskeletal assessment were excluded. Consideration of visceral, metabolic, or endocrine causes of low back pain, while important in broader clinical contexts, fell outside the scope of this sport-focused, cross-sectional study.^[1]^

Second, occupational and non-sport physical activity may influence the development of NSLBP. The study population consisted primarily of university-based amateur athletes with broadly comparable academic and training environments. Occupational workload was therefore not prioritised to maintain feasibility and focus on sport-specific variables. This limitation was acknowledged, and future studies incorporating objective workload monitoring across sport, occupation, and daily activity are warranted.

Third, the influence of medication use, diet, and substance exposure on NSLBP is recognised. These variables were not included due to the exploratory nature of the study, concerns regarding recall bias, and participant burden. Importantly, the omission of these factors does not imply that biomechanical contributors act in isolation; rather, they represent modifiable components within a broader multifactorial framework.

Fourth, we agree that different overhead sports impose distinct mechanical demands. This observation aligns with our findings, which demonstrated variation in NSLBP prevalence across overhead sports, with higher rates observed in volleyball and basketball compared to swimming and tennis. Similar sport-specific differences in spinal loading among overhead athletes have been reported previously.^[4]^

Finally, we acknowledge the influence of sleep quality, psychological stress, and psychosocial factors on NSLBP. These were partially addressed through the inclusion of the Keele STarT Back Screening Tool and further acknowledged in the discussion and future research recommendations. Comprehensive biopsychosocial modelling was not an objective of the present study but represents an important direction for future research.

In conclusion, this study provides clinically relevant insight into NSLBP prevalence and selected biomechanical risk factors in an under-researched South African amateur athletic population. I appreciate Dr Finsterer’s contribution to the scholarly dialogue and agree that continued research adopting broader, integrative models is needed.
